# Lightweight CycleGAN models for cross-modality image transformation and experimental quality assessment in fluorescence microscopy

**DOI:** 10.1364/BOE.578297

**Published:** 2026-02-18

**Authors:** Mohammad Soltaninezhad, Yashar Rouzbahani, Jhonatan Contreras, Francisco Paez Larios, Paul M. Jordan, Oliver Werz, Rohan Chippalkatti, Daniel Kwaku Abankwa, Christian Eggeling, Thomas Bocklitz

**Affiliations:** 1 Department “Photonic Data Science”, Leibniz Institute of Photonic Technology, Member of Leibniz Health Technologies, Member of the Leibniz Centre for Photonics in Infection Research (LPI), Jena, Germany; 2 Work group “Photonic Data Science”, Institute of Physical Chemistry (IPC) and Abbe Center of Photonics (ACP), Friedrich Schiller University Jena, Member of the Leibniz Centre for Photonics in Infection Research (LPI), Jena, Germany; 3 Institute of Applied Optics and Biophysics Friedrich Schiller University Jena, Jena, Germany; 4 Leibniz Institute of Photonic Technologies Department of Biophysical Imaging, Jena, Germany; 5 Department of Pharmaceutical/Medicinal Chemistry, Institute of Pharmacy, Friedrich Schiller University Jena, 07743 Jena, Germany; 6 Jena Center for Soft Matter (JCSM), Friedrich Schiller University Jena, 07743 Jena, Germany; 7 Cancer Cell Biology and Drug Discovery group, 2 Bioinformatics Core, Department of Life Sciences and Medicine, University of Luxembourg, L-4367 Esch-sur-Alzette, Luxembourg

## Abstract

With the growing integration of artificial intelligence in scientific and medical applications, lightweight deep learning models have become increasingly important. These models offer substantial reductions in memory usage and computational time. Given that GPU-based model training and inference contribute significantly to carbon emissions, lightweight architectures with comparable performance to parameter-rich models present a more environmentally friendly alternative. Specifically, we build upon CycleGAN with a fixed-channel lightweight U-Net generator for modality transfer from standard confocal to super-resolution STED and deconvolved STED images, and systematically compare it against Pix2Pix and standard CycleGAN baselines. Obtaining paired datasets in medical imaging and super-resolution microscopy is often infeasible due to the need for additional experiments and the intrinsic complexity of biological sample preparation. To address this, we investigate the performance of lightweight CycleGAN models, demonstrating their ability to achieve high-fidelity modality transfer despite reduced model complexity. We introduce a fixed channel strategy within the U-Net-based generator, in contrast to the traditional channel-doubling approach. This modification significantly reduces the number of trainable parameters from 41.8 million to approximately 9 thousand, while achieving comparable or slightly improved performance. We explore the utility of GAN models as a qualitative marker for assessing experimental and labeling quality. When trained on high-quality microscopy images, the GAN implicitly learns the characteristics of optimal imaging. Deviations between GAN-generated outputs trained on high-quality data and low-quality experimental images can highlight potential issues such as photobleaching, experimental artifacts, or inaccurate labeling. In this way, the model can support qualitative assessment of experimental consistency and image fidelity in fluorescence microscopy workflows.

## 1. Introduction

Fluorescence microscopy is an essential analysis tool in many biomedical and life science applications. Here, confocal microscopy provides enhanced imaging contrast over traditional light microscopy, allowing detailed imaging of cellular structures. However, its resolution is limited by the diffraction barrier, typically around 200 nm. Super-resolution microscopy, such as Stimulated Emission Depletion (STED) microscopy, surpasses this limitation by reversibly switching on and off the fluorescence emission [1]. In STED microscopy, a depletion laser is employed to selectively deplete the fluorescence emission of fluorophores at the focal periphery, achieving resolutions beyond the diffraction limit. Routinely, STED microscopes reach a standard resolution of approximately 30-60 nm in biological samples [1,2]. While STED microscopy offers superior spatial resolution, it presents experimental challenges such as photobleaching, phototoxicity, fluorescent labeling limitations, and experimental complexity. High-intensity depletion lasers in STED can lead to irreversible fluorophore degradation and cellular damage, limiting long-term imaging capabilities [3]. Moreover, the intricate setup and precise alignment required for STED increase its experimental demands. These limitations motivate the pursuit of computational approaches capable of replicating STED-level resolution without requiring direct super-resolution acquisition [4].

An often employed straightforward approach to potentially enhance spatial resolution in microscope images is deconvolution. Here, the image is deconvolved with the imaging or point-spread-function (PSF) of the microscope to reduce noise and enhance high spatial frequencies in the image. However, deconvolution comes with its own set of challenges and problems. Deconvolution relies heavily on high-quality microscopy images as input, as poor initial image quality can lead to errors in the final reconstructed image. One major challenge is noise amplification, where the deconvolution process enhances noise rather than resolving finer structural details, particularly in regions with low signal. This can create artifacts that obscure the true biological structures being analyzed. Computational complexity is another concern, as high-resolution microscopy images require significant processing power and time to perform deconvolution effectively. Advanced deconvolution methods can be resource-intensive, potentially slowing down analysis pipelines. Additionally, deconvolution accuracy depends on having a well-characterized PSF for the microscope; discrepancies between the assumed and actual PSF can result in distorted reconstructions [5]. Addressing these challenges requires careful optimization of imaging conditions, robust noise suppression techniques, and advanced computational algorithms specifically adapted for the microscopy technique in use. Meanwhile, artificial intelligence (AI) methods can help generate comparable images using Generative Adversarial Networks (GANs). GANs are increasingly used in medical imaging tasks such as image reconstruction, segmentation, synthesis, and cross-modality translation [6]. They have shown notable success in other imaging modalities, including Positron Emission Tomography (PET), Magnetic Resonance Imaging (MRI), and Computed Tomography (CT) by generating realistic data, improving image quality, and reducing domain shift [7]. In the field of microscopy, GANs have been employed to enhance fluorescence image quality, resolution, and modality conversion. They are particularly effective in denoising low-SNR images, enabling imaging under low illumination to minimize photobleaching and photodamage [8–12].

Studies have applied convolutional encoder–decoder networks and GAN-based approaches to enhance fluorescence microscopy, including denoising, super-resolution, and cross-modality translation between widefield fluorescence microscopy (WFM), confocal laser scanning microscopy (CLSM), stimulated emission depletion microscopy (STED), structured illumination microscopy (SIM), single-molecule localization microscopy (SMLM), and electron microscopy (EM) [13–19]. Pix2Pix, CycleGAN, and related architectures have been used to transform between imaging modalities (e.g., between CLSM and STED, from label-free to labeled images) and to reconstruct high-resolution or SMLM-like images from sparse or low-SNR data, illustrating the broad applicability of deep generative models in fluorescence and super-resolution microscopy [20–25].

Among these, Pix2Pix and CycleGAN are widely favored [26]. Pix2Pix uses a paired learning framework, while CycleGAN enables training via cycle-consistency loss, making it suitable for unpaired learning when aligned datasets are unavailable [27]. The cycle-consistency loss enforces that an image translated from one domain to another and then back should closely match the original. This is achieved using an inverse mapping that extracts information from both modalities, enhancing learning stability. Pix2Pix is simpler and less computationally intensive; CycleGAN, though more complex, on the other hand, offers greater flexibility, especially valuable in medical imaging and microscopy, where obtaining paired images is often challenging. It is important to note that a GAN consists of two core components: a generator, which attempts to create realistic images, and a discriminator, which aims to distinguish between real and generated images. These two networks are trained adversarially to improve each other’s performance over time. Generator networks like U-Net and ResNet are commonly used [25,28]. U-Net offers spatial detail preservation due to its skip connections and has demonstrated strong performance in modality transfer and super-resolution tasks [29,30].

Cycle-consistent transformation has been applied across microscopy tasks, as evidenced by a comprehensive review that synthesizes cross-modality transformations for contrast and resolution and highlights challenges in interpretability and validation [31]. One study reported that a cycle-consistent generative adversarial network (CycleGAN) framework achieved confocal super-resolution, translating low to high-resolution confocal images and outperforming prior baselines on peak signal-to-noise ratio (PSNR) and frequency-content metrics [32]. In pathology, an unsupervised cycle-consistent framework augmented with Multiple-Instance Learning (MIL) and conditioning signals generated Ki-67 immunohistochemistry (IHC) images from hematoxylin-and-eosin (H&E)–stained pathology images using unpaired images, showing clinical promise while underscoring the need for stronger evaluation and larger datasets [33]. A cycle-consistency–based translation approach from Microscopy with Ultraviolet Surface Excitation (MUSE) to formalin-fixed, paraffin-embedded (FFPE)–like images, combined with a classifier, improved metastasis detection by mapping unpaired images into a label-compatible domain [34]. More broadly, surveys of unsupervised/self-supervised deep learning (DL) motivate cycle-consistent methods precisely because paired ground truth is scarce and unpaired images dominate real-world datasets [35]. Across these studies, recurring challenges include interpretability and validation gaps, domain shift and limited generalization to unseen samples or instruments, dataset scale and data-quality coverage requirements, and risks of hallucination or morphology drift that can compromise biological fidelity. Lightweight CNN backbones such as SqueezeNet, MobileNet [36], and ShuffleNet have been explored to reduce computational cost, and MobileNet-like variants have been used in image-to-image translation outside of microscopy and medical imaging. However, these architectures are not specifically tailored for U-Net–style generators in fluorescence microscopy. Here, we investigate a fixed-channel lightweight U-Net generator within the CycleGAN framework for confocal-to-STED and deconvolved STED image translation [37,38].

This approach is particularly relevant for live-cell or dose-sensitive imaging, high-throughput or multi-site studies with limited STED microscopy access, and real-time use during image acquisition in resource-constrained settings.

We first directly compare Pix2Pix and CycleGAN on a co-registered paired dataset to assess the performance differences between paired and unpaired training under ideal conditions. We then evaluate a series of U-Net–based CycleGAN generator architectures, ranging from parameter-rich to lightweight models, for cross-modality image transformation from confocal to STED and deconvolved STED microscopy, and investigate their performance and robustness under experimental variability. We introduce a fixed-channel lightweight U-Net generator within the CycleGAN framework and systematically analyze nine generator variants in terms of reconstruction quality, parameter count, and inference cost [39,40]. This design substantially reduces trainable parameters and memory requirements, facilitates deployment in resource-constrained or microscope-integrated environments, and is expected to reduce energy consumption and associated CO_2_ emissions compared to heavier models [39–42]. Additionally, we explore GANs trained on high-quality STED and deconvolved STED images as a potential proxy to highlight possible experimental or labeling inconsistencies in lower-quality acquisitions, using ARL13B-labeled primary cilia as a model system [43]. Finally, we perform an exploratory evaluation of one trained CycleGAN model on an independent dataset of images of fluorescently stained mitochondria in fixed human primary M2 macrophages to assess its generalization behavior to unseen data.

## 2. Methods

### 2.1. Biological sample preparation and imaging acquisition

The dataset used in this study was derived from fluorescence microscopy imaging of ARL13B-labeled primary cilia. ARL13B is a small Ras-family GTPase anchored to the ciliary membrane and widely used as a marker for cilia structure and function. For confocal and STED microscopy, cilia were fluorescently labeled using a rabbit polyclonal anti-ARL13B primary antibody (177-11-1AP, Proteintech; 1:500), followed by a goat anti-rabbit Alexa Fluor 594 secondary antibody (A-11012, Thermo Fisher Scientific; 1:200), according to standard immunofluorescence protocols.

**Fig. 1.**
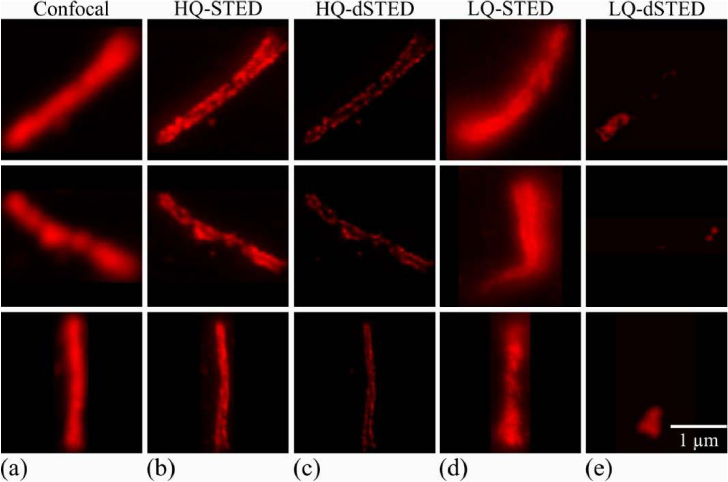
Representative datasets overview of confocal and differently processed images of immunolabeled ARL13B in primary cilia cells. Each row of panel columns a-c and of d-e presents a different sample: (a) confocal, (b) corresponding STED, (c) deconvolved STED, (d) low-quality STED, and (e) deconvolved low-quality STED images. All images feature the same spatial scale.

STED and confocal microscopy images were acquired on an Abberior Infinity Line microscope (Abberior GmbH, Göttingen, Germany) equipped with Imspector software version 16.3.16118-w2224 and an Olympus 60 × oil objective (1.42 NA), and using a 561 nm picosecond pulsed laser diode, a 775 nm pulsed STED laser (NKT Photonics Switzerland GmbH, Regensdorf, Switzerland), and an avalanche photodiode detector (Excelitas Technologies Corp., USA) with 5 µs dwell time and line averaging of 10. A total of 256 high-quality co-registered confocal–STED image pairs and 166 corresponding high-quality deconvolved STED images were acquired. Each set consists of confocal, STED, and deconvolved STED images obtained from the same field of view. Deconvolved STED images were generated from raw STED microscopy data using the Classic Maximum Likelihood Estimation (CMLE) algorithm implemented in Huygens Professional software (Scientific Volume Imaging, The Netherlands). The CMLE algorithm improves spatial resolution by iterative deconvolution based on the system point spread function. In addition to the high-quality dataset, an independent set of 48 low-quality STED and a set of 13 low-quality deconvolved STED images were acquired under sub-optimal imaging conditions to evaluate model robustness. This low-quality STED dataset was not used for training; the corresponding confocal images served as a test set. All datasets were verified for spatial co-registration and used for the deep learning experiments (Fig. 1). In addition, to assess cross-dataset generalization, we used an independent dataset of 42 paired confocal and STED images of mitochondria immunolabeled for Translocase of the Outer Mitochondrial Membrane 20 (TOM20) with Abberior STAR RED in human primary M2 macrophages, acquired under standard high-quality conditions. Human M2 macrophages were generated from leukocytes concentrates from healthy blood donors, the general protocol is described in Günther et al [44]. After fixation, M2 macrophages were fluorescently labeled using a rabbit polyclonal anti-TOM20 primary antibody (11802-1-AP, Proteintech; 1:100), followed by a goat anti-rabbit STAR RED secondary antibody (STRED-1002, Abberior; 1:200), according to standard immunofluorescence protocol. This TOM20 dataset was not used for training and served exclusively to evaluate the generalization performance of the selected lightweight CycleGAN model.

### 2.2. Image processing and augmentation

**Fig. 2.**
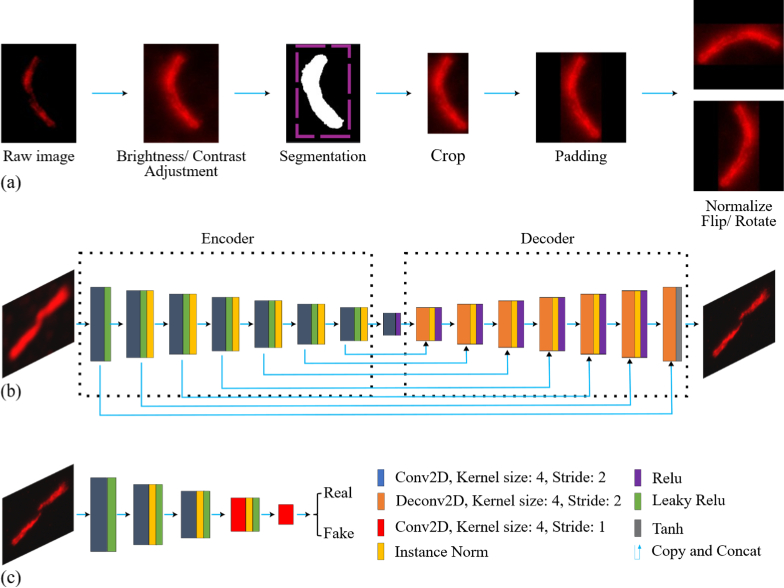
Image preprocessing workflow and GAN model architecture. (a) Schematic illustration of the image preprocessing and data augmentation pipeline. The workflow begins with brightness and contrast enhancement using intensity histogram normalization to standardize image brightness and contrast across samples. This is followed by segmentation and cropping of the cilium region of interest, then padding to a fixed size of 128 × 128 pixels. Finally, images are normalized and augmented through random rotations (in 90° increments) and horizontal/vertical flipping to improve model training robustness. (b, c) Architectures of the generator and discriminator networks. The generator is a U-Net with a symmetric encoder-decoder structure and skip connections, enabling preservation of spatial features across scales. The discriminator follows the PatchGAN design, evaluating overlapping image patches to distinguish real from generated images, thereby encouraging the generator to produce realistic local textures.

Prior to training, microscopy images required systematic preprocessing to ensure compatibility with the neural network input and to enhance image quality for model learning (see Fig. 2(a)). To ensure pixel-wise correspondence between modalities, confocal and STED images were rigidly co-registered using a mutual information–based alignment, with visually verified overlays. Image contrast was standardized using global Enhance Contrast operation in ImageJ applied uniformly to all images [45]. Full co-registration and contrast enhancement details are provided in the Supplement 1 S1. Segmentation was performed to isolate individual cilia and crop each field of view around the target region. To meet the input requirements of the U-Net–based architectures while preserving the original spatial resolution, the cropped images were padded to a standardized size of 128 × 128 pixels. Rescaling can cause artificial changes in pixel size or aspect ratio that distort morphology, whereas padding instead preserves structure and enables consistent down and up sampling within the networks [46]. The identical preprocessing pipeline was applied to all methods, including the confocal baseline, so that observed performance differences can be attributed to the learned image translation rather than to the preprocessing steps.

Before data augmentation, normalization was applied to standardize image intensity distributions across the dataset. For data augmentation, we applied only conservative geometric transformations to preserve biological structure. Specifically, random rotations (90°, 180°, 270°) and horizontal and vertical flipping were used to increase dataset variability while retaining the complete structural information of the cilium. No additional intensity or scale augmentations were applied to maintain the integrity of fluorescence signal distributions across the dataset.

### 2.3. GAN architectures and training strategy

We compared two generative adversarial network (GAN) architectures: Pix2Pix and CycleGAN. The generator network for all models was based on the U-Net architecture. The discriminator used a PatchGAN configuration to evaluate local image patches and discriminate between real and fake images [47].

Pix2Pix was applied with paired confocal and STED/deconvolved STED images. It minimizes a combination of L1 loss (Eq. (2)), to encourage similarity between generated and target images, and adversarial loss from the discriminator (Eq. (3)). The full Pix2Pix objective is given in Eq. (1) [25].

(1) $$\text{Los}s_{\text{Pix2Pix}}(G,D)=\text{Los}s_{\text{GAN}}(G,D)+\lambda \cdot \text{Los}s_{L1}(G)$$

(2) $$Loss_{L1}(G)=E_{x,y}[{||y-G(x)||}_{1}]$$

(3) $$\text{Los}s_{\text{cGAN}}(G,D)=E_{x,y}[\log D(x,y)]+E_{x}[\log(1-D(x,G(x)))]$$

Here, G and D denote the generator and discriminator networks, respectively, while x represents the input confocal image and y is the corresponding STED or deconvolved STED image. The symbol E represents the expectation over the training data, and ∥⋅∥ 1 indicates the L1 norm. The scalar λ balances the contribution of the adversarial loss and the L1 reconstruction loss.

For Pix2Pix, only the most complex generator architecture was used as a reference, whereas the full channel reduction study was conducted exclusively for CycleGAN models.

CycleGAN employs two coupled generator–discriminator networks to perform unpaired image-to-image translation. Its objective function combines an adversarial loss, a cycle-consistency loss to enforce reversibility between source and target domains, and an optional identity loss to encourage structural and intensity preservation when the source and target domains are similar. The total loss is defined in Eq. (4), with the cycle-consistency and identity components detailed in Eqs. (5) and (6), respectively [26].

(4) $$\text{Los}s_{\text{CycleGAN}}=\text{Los}s_{\text{GAN}}(G,D_{Y})+\text{Los}s_{\text{GAN}}(F,D_{X})+\lambda_{\text{cyc}}\cdot \text{Los}s_{\text{cyc}}(G,F)+\lambda_{\text{id}}\cdot \text{Los}s_{\text{id}}(G,F)$$

(5) $$\text{Los}s_{\text{cyc}}(G,F)=E_{x}[{\left|F(G(x))-x\right|}_{1}]+E_{y}[{\left|G(F(y))-y\right|}_{1}]$$

(6) $$\text{Los}s_{\text{id}}(G,F)=E_{y}[{\left|G(y)-y\right|}_{1}]+E_{x}[{\left|F(x)-x\right|}_{1}]$$

In these equations, F denotes the inverse generator mapping from the target domain to the source. D_X_ and D_Y_ are the discriminators for the source and target domains, respectively. The scalar λ_cyc_ controls the importance of the cycle-consistency loss, while λ_id_ weights the identity loss. The term Loss_cyc_ ensures that mappings are reversible (i.e., x→y→x), and Loss_id_ encourages generators to behave like identity mappings when source and target domains are aligned, such as in STED → STED translation.

To investigate the influence of model complexity on performance, we designed a series of generator variants with systematically reduced numbers of feature channels. Two distinct architectural strategies were implemented: in the doubling-channel U-Net design, channels double at each down-sampling stage while the initial channel count is progressively halved across variants (from 64 with a 512-channel bottleneck down to 8 with a 64-channel bottleneck; Models 1–4 and 6). In the fixed-channel U-Net design, every layer uses the same number of channels, and this constant is reduced across variants (64, 32, 16, and 8; Models 5 and 7–9). The model index increases as the architectural complexity decreases. All models were trained on the co-registered confocal and STED dataset under identical conditions to ensure consistency across experiments (Fig. 2(b), (c)). A complete list of hyperparameters (learning rate, batch size, optimizer, weights loss, etc.) and training settings is provided in the Supplement 1 S2, together with representative training and validation loss curves, variance across random seeds, and example failure cases illustrating the effect of our regularization choices on training stability.

### 2.4. Computing & evaluation

Training and evaluation were performed on an NVIDIA GeForce RTX 4090 (24 GB). For each experiment, we reinitialized the pipeline (cleared GPU cache, reset random seeds across libraries, removed prior checkpoints) to ensure reproducibility and used 5-fold cross-validation, with each image appearing in the validation set once. Image quality was assessed using SSIM, PSNR, and FRC, reported as mean ± standard deviation across folds. Statistical comparisons between models were performed using repeated-measures ANOVA with images as subjects and model as the within-factor, followed by Holm-corrected pairwise tests for multiple comparisons. Formal metric definitions, implementation details, GPU/protocol settings, and full cross-validation splits are provided in the Supplement 1 S3.

## 3. Results and discussion

### 3.1. Pix2Pix vs. CycleGAN

Using differently trained algorithms, we aimed at cross-modality image translation from confocal to STED and deconvolved STED microscopy images. As an experimental example, we took multiple sets of confocal and STED microscopy images of immunolabeled ARL13B at primary cilia cells. We started with CycleGAN and Pix2Pix algorithms.

**Fig. 3.**
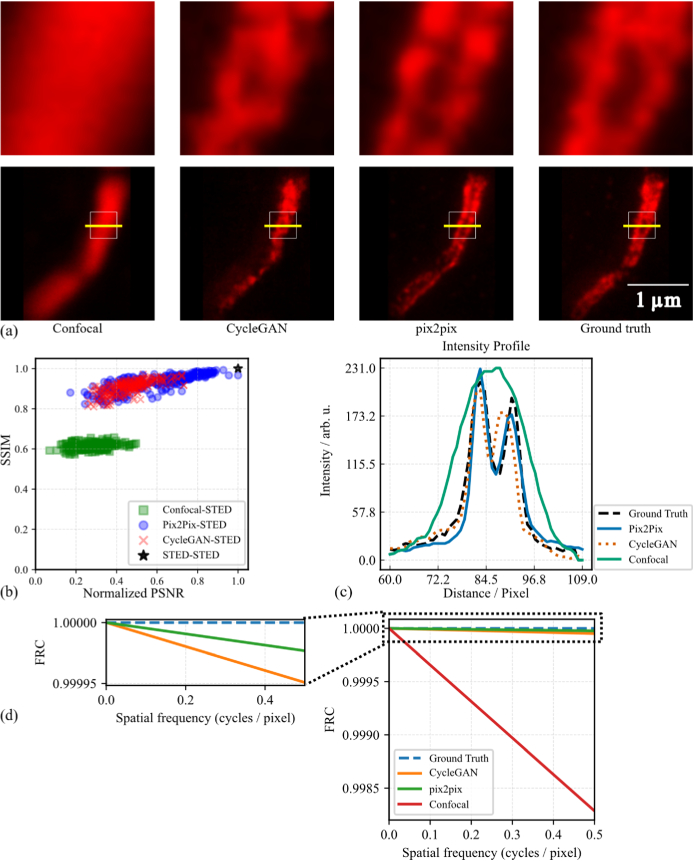
Comparison between Pix2Pix and CycleGAN methods for Confocal-to-STED cross modality Transfer. (a) Representative images of the confocal to STED translation task. From left to right: input confocal image, CycleGAN-generated STED image, Pix2Pix-generated STED image, and the STED ground truth. White boxes indicate regions shown in the zoomed-in views, and yellow lines mark the positions used for intensity profile analysis. (b) Comparison of image quality metrics (SSIM vs. normalized PSNR) for Pix2Pix-generated, CycleGAN-generated, and Confocal images. Each point represents an individual test sample, with marker color and shape indicating the method. The black star at (1.0, 1.0) denotes the STED-STED reference. (c) Intensity profiles along the yellow lines in (a), comparing the confocal input, CycleGAN and Pix2Pix outputs, and the ground truth STED image. All images feature the same spatial scale. (d) Fourier ring correlation (FRC) analysis of the field of view (FOV) with respect to the STED ground truth.

We first evaluated the performance of CycleGAN and Pix2Pix using the paired confocal and STED microscopy images. With paired data, we have known matches from both modalities as ground truth, which is not available in the unpaired scenario. Using paired data to test CycleGAN is an effective strategy to assess its performance in settings where ground truth is otherwise unavailable. In our experiment, Pix2Pix slightly outperformed CycleGAN. However, CycleGAN still achieved competitive results. Across the entire dataset, for the confocal to STED modality transfer task, Pix2Pix achieved an average SSIM of 0.93 ± 0.04 and a normalized PSNR of 0.61 ± 0.18, both computed using the ground truth STED images as reference. In comparison, CycleGAN reached an SSIM of 0.90 ± 0.04 and a normalized PSNR of 0.46 ± 0.10, while the confocal baseline had an SSIM of 0.60 ± 0.02 and a normalized PSNR of 0.27 ± 0.09 (Fig. 3(b)). Figure 3(a) presents an example of the confocal-to-STED modality transfer task. From left to right, the images show the input confocal image, CycleGAN-generated STED, Pix2Pix-generated STED, and the ground truth (real) STED image.

In Fig. 3(c), the intensity profile is plotted along the yellow line shown in the images in Fig. 3(a). Based on the extracted intensity profiles, both Pix2Pix and CycleGAN demonstrate the ability to reconstruct structural features from the confocal input, with varying degrees of fidelity. Notably, both models recover the presence of two distinct peaks, while the confocal profile resembles a single broad Gaussian-like shape. The Pix2Pix generation closely follows the STED ground truth, with a slight tendency to overestimate peak intensities, whereas CycleGAN slightly underestimates them. In contrast, the confocal profile exhibits broader and less defined peaks, consistent with its lower resolution. These observations are supported by Pearson correlation coefficients with the ground truth profile: Pix2Pix (0.976), CycleGAN (0.927), and confocal (0.893). Consistent with this, Fourier ring correlation (FRC) with respect to the STED ground truth (Fig. 3(d)) showed identical FRC-based resolution for all methods. Reporting high-frequency disagreement as 1−FRC , the mean values were 0 (ground truth), $5\times 10^{-5}$ (CycleGAN), $2\times 10^{-5}$ (Pix2Pix), and $1.71\times 10^{-3}$ (confocal), indicating that the AI-generated images closely match the ground truth in the frequency domain and outperform the confocal input, with Pix2Pix performing slightly better than CycleGAN.

**Fig. 4.**
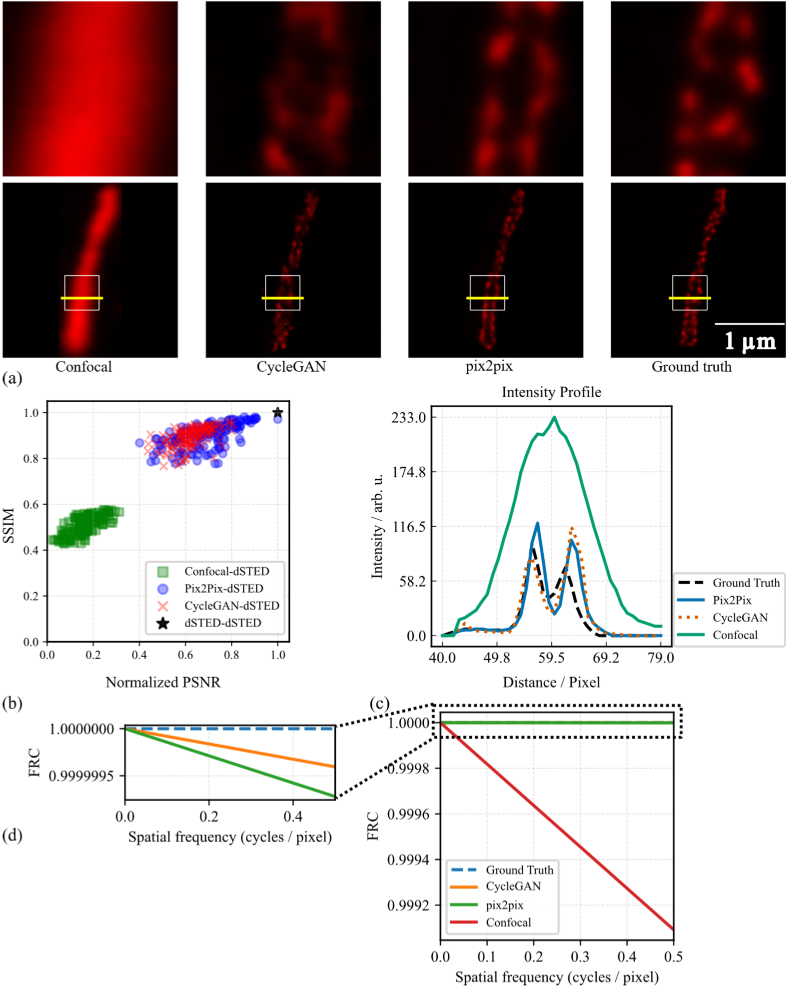
Comparison between Pix2Pix and CycleGAN methods for confocal to deconvolved STED (dSTED) cross modality transfer. (a) Representative images of the confocal to dSTED translation task. From left to right: input confocal image, CycleGAN-generated dSTED image, Pix2Pix-generated dSTED image, and the dSTED ground truth. White boxes indicate regions shown in the zoomed-in views, and yellow lines mark the positions used for intensity profile analysis. (b) Comparison of image quality metrics (SSIM vs. normalized PSNR) for Pix2Pix-generated, CycleGAN-generated, and Confocal images. Each point represents an individual test sample, with marker color and shape indicating the method. The black star at (1.0, 1.0) denotes the dSTED-dSTED reference. (c) Intensity profiles along the yellow lines in (a), comparing the confocal input, CycleGAN and Pix2Pix outputs, and the ground truth dSTED image. All images feature the same spatial scale. (d) Fourier ring correlation (FRC) analysis of the field of view (FOV) with respect to the dSTED ground truth.

For the confocal-to-deconvolved STED (dSTED) task, across the entire dataset, average SSIM and normalized PSNR values across the test dataset show Pix2Pix (SSIM: 0.89 ± 0.05, PSNR: 0.69 ± 0.11) outperforms CycleGAN (SSIM: 0.88 ± 0.05, PSNR: 0.62 ± 0.07) and the confocal input (SSIM: 0.50 ± 0.04, PSNR: 0.17 ± 0.07), as shown in Fig. 4(b).

Figure 4(a) shows, from left to right, the confocal input, CycleGAN output, Pix2Pix output, and the ground truth dSTED image. Both models aim to generate the corresponding dSTED modality from the confocal input. In Fig. 4(c), the intensity profile extracted along the yellow lines in Fig. 4(a) illustrates the ability of the models to recover fine structural features from the confocal input. Both Pix2Pix and CycleGAN successfully reconstruct the two-peak structure and capture the overall profile shape of the ground truth. However, both models overestimate the peak intensities, with CycleGAN slightly exaggerating the second peak more prominently. The confocal input shows a broadened signal with reduced resolution. These observations are reflected in the Pearson correlation coefficients with the ground truth: Pix2Pix (0.868), CycleGAN (0.823), and confocal (0.803), highlighting the improved structural fidelity achieved by the GAN-based models. FRC of the same FOV with respect to the dSTED ground truth (Fig. 4(d)) showed identical FRC-based resolution for all methods. Reporting high-frequency disagreement as 1−FRC , the mean values were 0 (ground truth), $2\times 10^{-7}$ (CycleGAN), $4\times 10^{-7}$ (Pix2Pix), and $6\times 10^{-7}$ (confocal), indicating that the

AI-generated images are highly similar to the ground truth in the frequency domain and outperform the confocal input, with CycleGAN performing slightly better than Pix2Pix in this case. These results highlight the comparative strengths and limitations of Pix2Pix and CycleGAN in fluorescence microscopy modality transfer tasks. While Pix2Pix outperforms CycleGAN in SSIM, PSNR, and intensity profile fidelity, its dependence on paired data, limits its applicability in many real-world scenarios. CycleGAN, although slightly less accurate, demonstrates strong reconstruction capabilities without requiring exact input-output alignment, making it a robust alternative when paired data are unavailable. The large performance gap between the confocal baseline and both GAN models also underscores the effectiveness of deep learning-based cross-modality translation in enhancing resolution and contrast.

### 3.2. Evaluating CycleGAN robustness with decreased network complexity

**Fig. 5.**
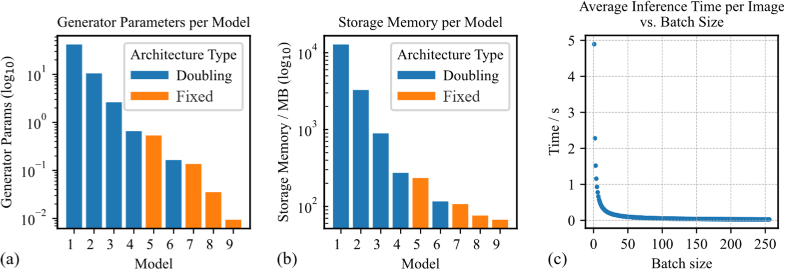
Summary of model complexity and performance characteristics across GAN architectures, including generator parameter count and storage memory (in megabytes). Inference time is reported for Model 1. (a) Number of parameters in the generator networks across all nine models. Models using a doubling channel strategy are shown in blue; those using a fixed channel strategy are shown in orange. The doubling architecture starts with 64 channels and reaches a bottleneck of 512 in Model 1 (41.8 M parameters), down to Model 6, which starts with 4 channels and has a bottleneck of 32. The fixed channel architecture begins with 64 channels in Model 5 and decreases to 8 channels in Model 9 (9000 parameters). For the number of parameters, see Table 1. (b) Storage usage for different models over 200 epochs, with checkpoints saved every 5 epochs (Table 1). (c) Per-image inference time across batch sizes for Model 1. Time is computed as the total runtime divided by the number of images.

We evaluated the modality transfer performance for both confocal to STED and confocal to deconvolved STED data using GAN models. The architecture is progressively simplified in complexity, with the number of parameters decreasing from 41.8 million in Model 1 to approximately 9 thousand in Model 9 (Fig. 5(a)).

**Table 1.** Model’s characteristics

Model	1	2	3	4	5	6	7	8	9
Generator Parameters (M)	41.8	10.45	2.61	0.65	0.53	0.16	0.13	0.035	0.009
Storage Memory (MB)	12800	3270	891	273	234	116	107	76	67

Correspondingly, the storage size of the saved weight decreases from 12.8 GB to 67 MB; all models were trained for 200 epochs, with model checkpoints saved every 5 epochs (Fig. 5(b)).

Table 1 summarizes the generator parameter counts and storage requirements, enabling a direct comparison of model efficiency, scalability, and performance across architectures with varying capacities. The inference time was measured using the largest model, with results shown in Fig. 5(c) and Table 2. As the number of test images increased from 1 to 256, the average inference time per image dropped from 4.89 s to 0.0255 s. This behavior is consistent with observations in GPU computing literature, where fixed overheads such as kernel launch, memory transfer, and synchronization dominate at small batch sizes but have diminishing impact at larger batch sizes [48]. Additionally, larger batches enable better parallelism and more efficient use of GPU cores, reducing idle time and maximizing throughput [49].

**Table 2.** Inference time per image

Number of images	1	25	50	100	150	200	250
Time (Seconds)	4.89	0.19	0.097	0.053	0.038	0.030	0.025

**Fig. 6.**
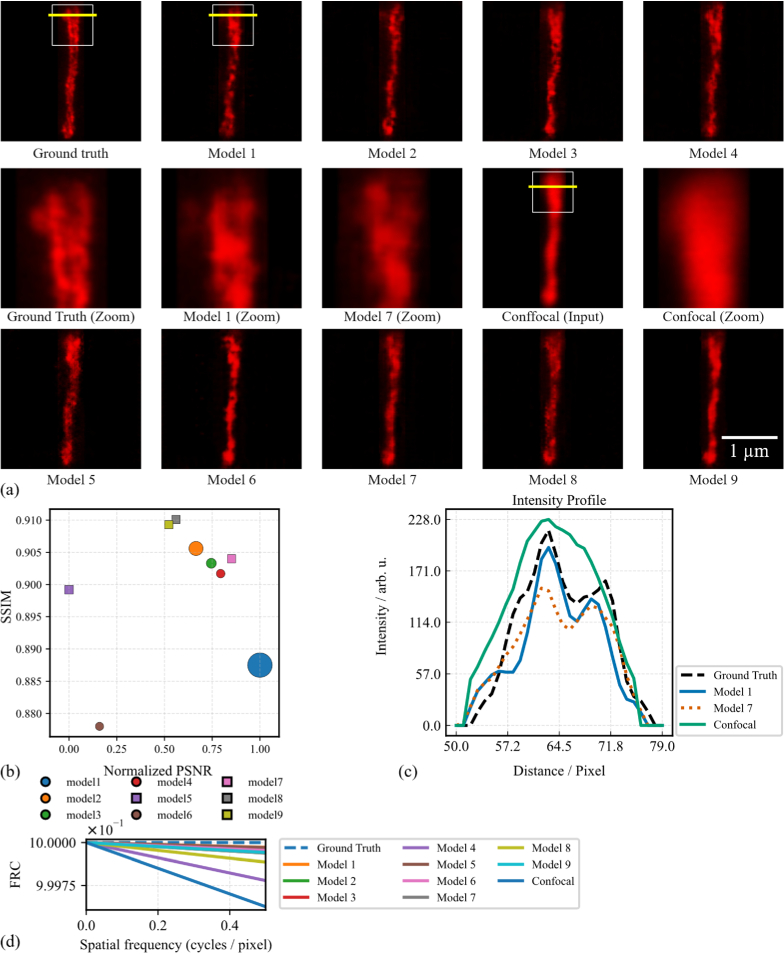
Evaluation of nine CycleGAN models for confocal-to-STED transfer. (a) Qualitative results for Models 1-9 vs. ground truth. Zoomed-in views show structural fidelity; yellow lines mark profile analysis areas. (b) Quantitative results (mean PSNR vs. SSIM) for all models, with marker shape/size indicating channel policy and parameter count. (c) Intensity profiles from (a) comparing Model 1, Model 7, confocal input, and ground truth. All images share the same spatial scale. (d) FRC analysis of models against the STED ground truth.

In the confocal-to-STED modality transfer task, image quality metrics across the nine GAN models represent averages computed over the entire test dataset and demonstrate a high degree of consistency. The highest SSIM value of 0.91 ± 0.041 is achieved by Model 8, while the lowest is 0.878 ± 0.035 in Model 6. For PSNR, the maximum value of 26.62 ± 2.07 dB is observed in Model 1, and the minimum is 26.11 ± 1.94 dB in Model 5. While the overall results are tightly clustered, slight performance differences are evident. Specifically, Models 1 and 8 exhibit slightly superior metrics in PSNR and SSIM, respectively, indicating marginally better structural and perceptual fidelity. These observations are further illustrated in Fig. 6(b) and quantitatively summarized in Table 3, which supports the robustness of the cross-modality transfer performance across varying model architectures. Repeated measures ANOVA revealed the main effect of models for both PSNR and SSIM (p < 0.001) Holm-corrected pairwise comparisons showed that most model pairs, including the largest model (Model 1) and the lightweight models (Models 7–9), did not differ significantly in PSNR, while a smaller subset of comparisons involving Models 4–6 (Model 1 vs 5, Model 4 vs 6, Model 5 vs 8 and 9) exhibited small but systematic differences (p < 0.001). SSIM-based tests indicated a similar pattern: structural fidelity was largely comparable across models, with broader but still modest differences concentrated around Models 5 and 6, whereas many other combinations (Models 1 vs 2, 3 vs 4, 7 vs 9) remained statistically indistinguishable (Supplementary Tables S3.1 and S3.2). For the Fourier ring correlation (FRC) analysis, fixed-channel models (5, 7, 8, and 9) clearly outperform the confocal baseline and substantially reduce high-frequency error; in particular, Models 5 and 9 achieve values comparable to the best parameter-rich Models 1 and 3 (see Table 7).

**Table 3.** Average Image Quality Metrics Across Models for Confocal to STED modality transfer (Full Dataset)

Model	1	2	3	4	5	6	7	8	9

SSIM	0.887	0.905	0.903	0.901	0.899	0.878	0.904	0.910	0.909
	±0.04	±0.04	±0.04	±0.042	±0.043	±0.035	±0.041	±0.041	±0.047

PSNR (dB)	26.62	26.45	26.49	26.51	26.11	26.19	26.54	26.39	26.37
	±2.07	±2.18	±2.14	±2.21	±1.94	±1.97	±2.13	±2.04	±2.09

**Fig. 7.**
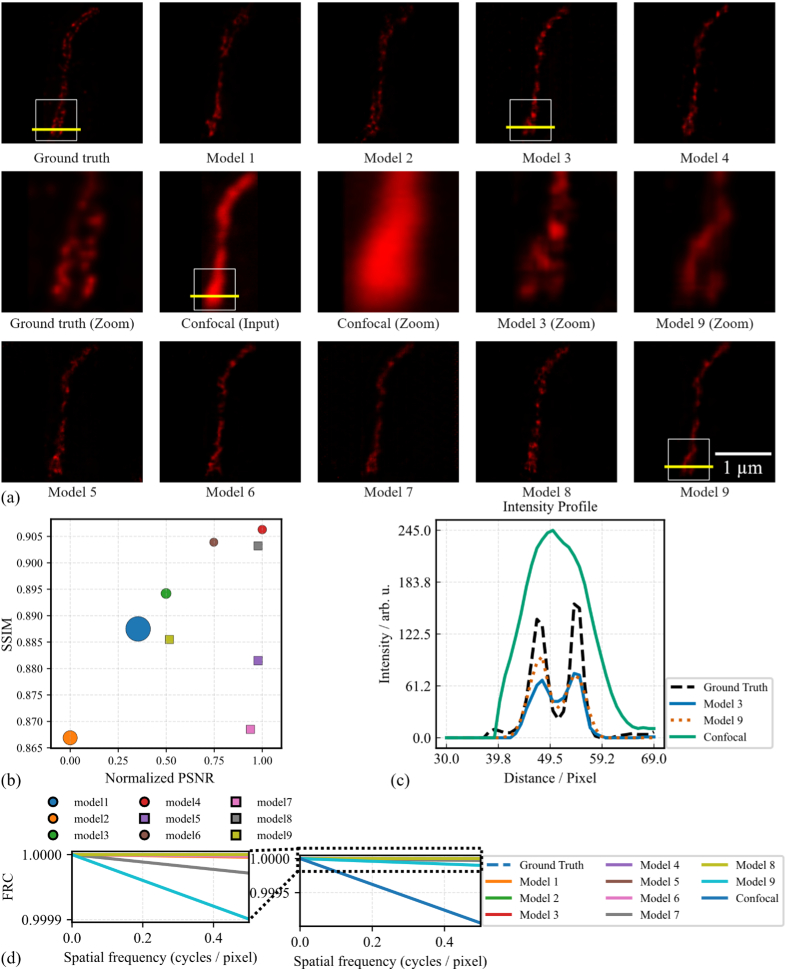
Evaluation of nine CycleGAN models for confocal to deconvolved STED (dSTED). (a) Qualitative results for Models 1-9 vs. ground truth dSTED. Zoomed-in views highlight structural fidelity; yellow lines mark profile analysis areas. (b) Quantitative results (mean PSNR vs. SSIM) for all models, with marker shape/size indicating channel policy and parameter count. (c) Intensity profiles from (a) comparing Model 3, Model 9, confocal input, and ground truth. All images share the same spatial scale. (d) FRC analysis of models against the deconvolved STED ground truth.

In the confocal-to-deconvolved STED modality transfer task, image quality metrics across the nine GAN models again show strong consistency, with all values representing averages computed over the entire test dataset. SSIM ranges from a minimum of 0.866 ± 0.073 (Model 2) to a maximum of 0.906 ± 0.058 (Model 4). PSNR values range from 26.25 ± 0.14 dB (Model 2) to a peak of 27.29 ± 0.15 dB (Model 4), suggesting improved reconstruction quality in the latter. Although the metric values are generally close across models, slight performance advantages can be observed. In particular, Model 4 achieves the best results across both metrics, with Models 6, 7, and 8 also performing competitively, indicating superior structural preservation and perceptual quality. These differences, though subtle, are evident in Fig. 7(b) and are quantitatively detailed in Table 5, reinforcing the robustness and consistency of the GAN-based modality transformation pipeline. Repeated-measures ANOVA again revealed a significant main effect of model on both SSIM and PSNR (p < 0.001). Holm-corrected pairwise SSIM comparisons showed that many model pairs, including the heavy architectures (Models 1–3) and the lightweight models (Models 7–9), were statistically similar, with only a subset of comparisons (notably involving Models 2, 4, 6, 8, and 9) showing modest but detectable differences. PSNR-based tests were more sensitive and yielded significant p-values for most pairs, yet the absolute PSNR differences remained small and did not consistently favor the largest networks, indicating that the lightweight, high-index models achieve intensity fidelity that is broadly comparable to that of the most parameter-rich architectures (Supplementary tables S3.3 and S3.4). For the FRC analysis, fixed-channel models (5, 7, 8, and 9) clearly outperform the confocal baseline and substantially reduce high-frequency error; in particular, Models 5 and 8 achieve values comparable to or even better than the most accurate parameter rich Models 1 and 2, while even Models 7 and 9 remain far below the confocal input, indicating that frequency-domain performance is largely preserved despite the strong reduction in model complexity (see Table 8). For more information about deviation maps and anomaly detection scores for the results presented in Figs. 6 and 7, see Supplement 1 S4. Overall, models using the fixed-channel policy, particularly Models 5, 7, 8, and 9, demonstrate that simpler, uniformly scaled architectures can perform competitively with more complex designs in cross-modality transfer tasks. These models provide strong reconstruction quality with substantially fewer parameters and reduced computational demand, indicating that fixed-channel designs offer an effective compromise between architectural simplicity and image quality. This observation is consistent with prior work linking more uniform capacity distributions to improved optimization behavior and training stability in deep networks [50,51]. While lighter architectures show strong performance, they remain at risk of losing fine structural details. Model 8 stands out among the fixed-channel architectures, achieving the highest SSIM in the confocal-to-STED task while maintaining competitive PSNR values. This indicates that a balanced, moderately lightweight design can provide both parameter efficiency and strong reconstruction quality. Nevertheless, subtle differences in structural detail and sharpness remain, as seen in the example outputs from all nine models in Fig. 6(a) and Fig. 7(a) for the confocal-to-STED and confocal-to-deconvolved STED transformations, respectively. Continuing with the confocal-to-STED example, image quality metrics were computed for a single representative sample across all models. The SSIM values range from 0.93 to 0.98, with Model 9 achieving the highest score, indicating strong structural similarity to the ground truth. PSNR varies from 28.8 dB (Model 5) to 32.1 dB (Model 7), with Models 4, 7, 8, and 9 exceeding 30 dB, reflecting improved noise suppression and intensity accuracy. These results suggest that while most models deliver high perceptual and structural quality on this sample, Model 7 stands out for the highest PSNR, and Model 9 achieves the best SSIM. Model 4 also performs consistently well across all three metrics. In contrast, Models 2 and 5 exhibit slightly lower performance, especially in PSNR. Overall, these sample-level metrics reinforce the earlier trend: models employing a fixed channel policy (e.g., 7, 9) tend to offer a favorable balance between image quality and architectural simplicity. The numerical values are detailed in Table 4, corresponding to the visual outputs in Fig. 6(a). In Fig. 6(c), the intensity profile is plotted along the yellow line shown in the images in Fig. 6(a). Model 1 (41.8 M parameters) and Model 7 (0.13 M parameters) both reconstruct the two-peak structure from the confocal input, capturing key spatial features present in the STED ground truth. Model 7 aligns closely with the ground truth in both peak position and relative intensity, while Model 1 shows a slight underestimation of the second peak. The confocal profile remains broader, with less distinct peak separation, consistent with its lower resolution. These observations are supported by Pearson correlation coefficients with the ground truth profile: Model 7 (0.9715), Model 1 (0.9411), and confocal (0.94). For the confocal-to-deconvolved STED modality transfer example shown in Fig. 7(a), image quality metrics were computed for a single representative sample across all nine GAN models. SSIM values are tightly grouped, with most models scoring 0.94, except Models 4, 7, and 8, which score slightly lower at 0.93 and 0.92, indicating minimal structural variation among outputs. PSNR values range from 31.0 dB (Model 8) to 33.3 dB (Model 9), with Models 7 and 9 achieving the highest scores, reflecting superior noise reduction and intensity fidelity. While all models deliver comparably high image quality on this sample, Model 9 stands out with the highest PSNR and SSIM scores, indicating excellent performance in both structure and contrast preservation. Model 7 also performs well. On the other hand, Model 8 shows slightly lower performance in PSNR and SSIM, suggesting minor degradation in sharpness or intensity. These detailed metrics are presented in Table 6 and visually supported by the example reconstructions shown in Fig. 7(a), providing further insight into architectural performance on a per-sample basis.

**Table 4.** Image Quality Metrics for the Sample Shown in Fig. 6(a) (Confocal to STED Transfer)

Model	1	2	3	4	5	6	7	8	9
SSIM	0.95	0.93	0.94	0.94	0.95	0.93	0.95	0.96	0.98
PSNR (dB)	29.5	28.9	30.3	31.6	28.8	29.4	32.1	30.8	31.8

**Table 5.** Average Image Quality Metrics Across Models for Confocal to Deconvolved-STED modality transfer (Full Dataset)

Model	1	2	3	4	5	6	7	8	9

SSIM	0.887	0.866	0.894	0.906	0.881	0.903	0.868	0.903	0.885
	±0.058	±0.073	±0.062	±0.058	±0.052	±0.057	±0.048	±0.057	±0.054

PSNR (dB)	26.62	26.25	26.77	27.29	27.26	27.02	27.22	27.26	26.79
	±0.13	±0.14	±0.14	±0.15	±0.14	±0.15	±0.16	±0.16	±0.17

**Table 6.** Image Quality Metrics for the Sample Shown in Fig. 7(a) (Confocal to deconvolved-STED Transfer)

Model	1	2	3	4	5	6	7	8	9
SSIM	0.94	0.94	0.94	0.93	0.94	0.94	0.92	0.92	0.94
PSNR (dB)	31.6	32.6	31.6	32.3	32.1	32.7	33.3	31	33.3

**Table 7.** Mean high-frequency FRC for the sample in Fig. 6(a), reported as 1−mean high - frequency for all models and the confocal image with respect to the STED image as ground truth (values in units of 10^−5^; lower values indicate better agreement).

Model	1	2	3	4	5	6	7	8	9	Confocal
FRC	2.8	6.1	4.1	22.2	3	4.7	6.2	11.4	5.8	37.5

**Table 8.** Mean high-frequency FRC for the sample in Fig. 7(a), reported as 1−mean high - frequency for all models and the confocal image with respect to the STED image as ground truth (values in units of 10^−6^; lower values indicate better agreement).

Model	1	2	3	4	5	6	7	8	9	Confocal
FRC	3.9	0.3	2	0.7	0.4	2.5	28.4	0.1	99.1	944.7

In Fig. 7(c), the intensity profile is plotted along the yellow line shown in the images in Fig. 7(a). Both Model 3 and Model 9 recover the two-peak structure present in the ground truth STED image, while the confocal profile remains broad and unresolved. Model 9, despite having significantly fewer parameters (0.009 M vs. 2.61 M in Model 3), achieves slightly better alignment with the ground truth in terms of peak position and separation. These trends are reflected in the Pearson correlation coefficients with the ground truth profile: Model 9 (0.929), Model 3 (0.923), and confocal (0.731).

On average, the CycleGAN models demonstrated slightly better SSIM scores in the confocal-to-STED modality transfer, while the confocal-to-deconvolved STED task yielded higher PSNR values. For the STED dataset, across all models, the mean SSIM and PSNR values were 0.90 and 26.41 dB, respectively. In contrast, the deconvolved STED dataset showed averages of 0.88 for SSIM and 26.94 dB for PSNR. These results suggest that while STED images preserve more structural similarity and perceptual detail, deconvolved STED images provide better intensity fidelity due to improved noise suppression [52]. This difference stems from the nature of the target data: deconvolved STED images have higher signal-to-noise ratios [53,54]. The improved image quality facilitates more stable training and better intensity accuracy. Thus, each modality presents unique challenges and advantages that influence the performance of GAN-based image translation models. In addition to channel configuration, further reductions in model complexity can be achieved by implementing depth-wise separable convolutions, which decompose standard convolutions into lighter operations, and by reducing network depth, thereby limiting the number of layers and associated parameters. These strategies help retain image fidelity while minimizing memory footprint and computational cost.

Moreover, in GAN-based image translation, various hyperparameters and architectural design choices substantially influence model behavior and performance. These include the type of generator architecture, the formulation of adversarial and reconstruction losses, and the choice of normalization strategies. Selecting appropriate convolutional kernel sizes based on the spatial scale of the target structures and input image resolution is also critical for effective feature extraction. Training dynamics are further affected by factors such as learning rate scheduling, weight initialization schemes, and the use of regularization techniques like dropout. These elements collectively shape model stability, convergence, and robustness, particularly when adapting across datasets with differing noise characteristics and structural complexity. Lighter models make it substantially easier to test these variations systematically. Due to their reduced training time and memory consumption, such models enable rapid experimentation, hyperparameter optimization, and cross-validation across diverse datasets.

Importantly, they also lower the computational burden, leading to reduced energy usage and significantly lower CO_2_ emissions, contributing to more environmentally sustainable deep learning practices. As deep learning models continue to scale, these considerations become increasingly relevant—not only for technical optimization but also for responsible and resource-aware research.

### 3.3. GAN-based inference as a qualitative benchmark for experimental and labeling fidelity

**Fig. 8.**
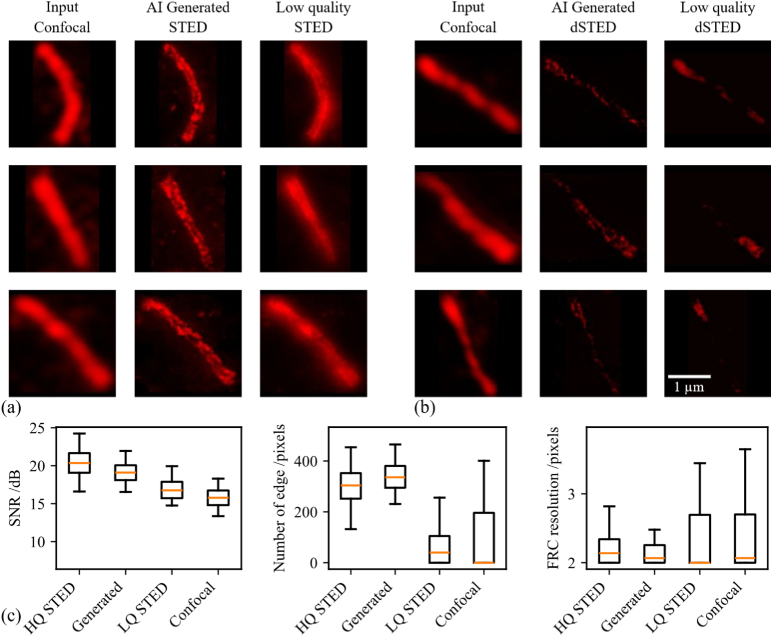
GAN-generated reconstructions as benchmarks for experimental quality. (a) Comparison of a confocal input, a GAN-generated STED image (trained on high-quality data), and a low-quality experimental STED image. (b) Similar comparison for deconvolved STED (dSTED). All images share the same spatial scale. (c) Quantitative comparison of high-quality STED, AI-generated STED, low-quality STED, and confocal images using signal-to-noise ratio, edge preservation, and FRC resolution on the whole dataset.

In Fig. 8(a), we evaluated the utility of a GAN model trained exclusively on high-quality STED data to serve as a reference for assessing experimental integrity. The model was trained to transform confocal input images into their corresponding high-resolution STED representations. When applied to confocal data, the GAN-generated outputs were compared to experimentally acquired low-quality STED images, which have been compromised due to suboptimal imaging conditions, photobleaching, or inaccurate protein labeling. The discrepancy between the GAN output and the degraded STED data highlights the sensitivity of the model to subtle experimental deviations. In Fig. 8(b), the same approach was applied to a confocal to deconvolved transformation task. Here, the GAN was trained with high-quality deconvolved images and tested on corresponding confocal images. The results presented in Fig. 8 demonstrate the potential of GAN-based models as benchmarks for evaluating experimental and labeling fidelity. A model trained with high-quality data captures an implicit standard of optimal structural features. Deviations between GAN predictions and new experimental outputs can indicate compromised imaging conditions or biological variability. This strategy is applicable across both STED and deconvolved STED modalities and enables indirect quality control in workflows where direct validation is limited. GAN-based inference can therefore assist in qualitatively identifying potential inconsistencies in sample preparation, imaging protocols, or labeling accuracy. To assess image quality without ground-truth references, we compared non-reference metrics such as signal-to-noise ratio (SNR), edge preservation, and Fourier ring correlation (FRC) based resolution across high-quality STED, AI-generated STED, low-quality STED, and confocal datasets. The AI-generated STED images closely approach the quantitative characteristics of the high-quality STED data: SNR (20.32 ± 1.72 for HQ-STED vs. 18.71 ± 2.71 for AI-generated), edge preservation (299.68 ± 88.86 vs. 338.00 ± 60.83), and FRC resolution (2.20 ± 0.20 px vs. 2.14 ± 0.15 px). In contrast, low-quality STED and confocal images show markedly lower SNR and edge preservation, together with higher FRC variability (2.34 ± 0.47 px for LQ-STED and 2.42 ± 0.58 px for confocal). The larger FRC standard deviations indicate unstable and inconsistent resolution performance in the LQ-STED and confocal datasets, whereas the AI-generated STED maintains resolution consistency comparable to the high-quality STED reference.

Since no ground-truth STED images exist for these samples, reference-based metrics cannot be applied here; however, the non-reference analysis demonstrates that the AI-generated outputs most closely mimic the quantitative behavior of high-quality STED images.

### 3.4. Generalization to an independent dataset

**Fig. 9.**
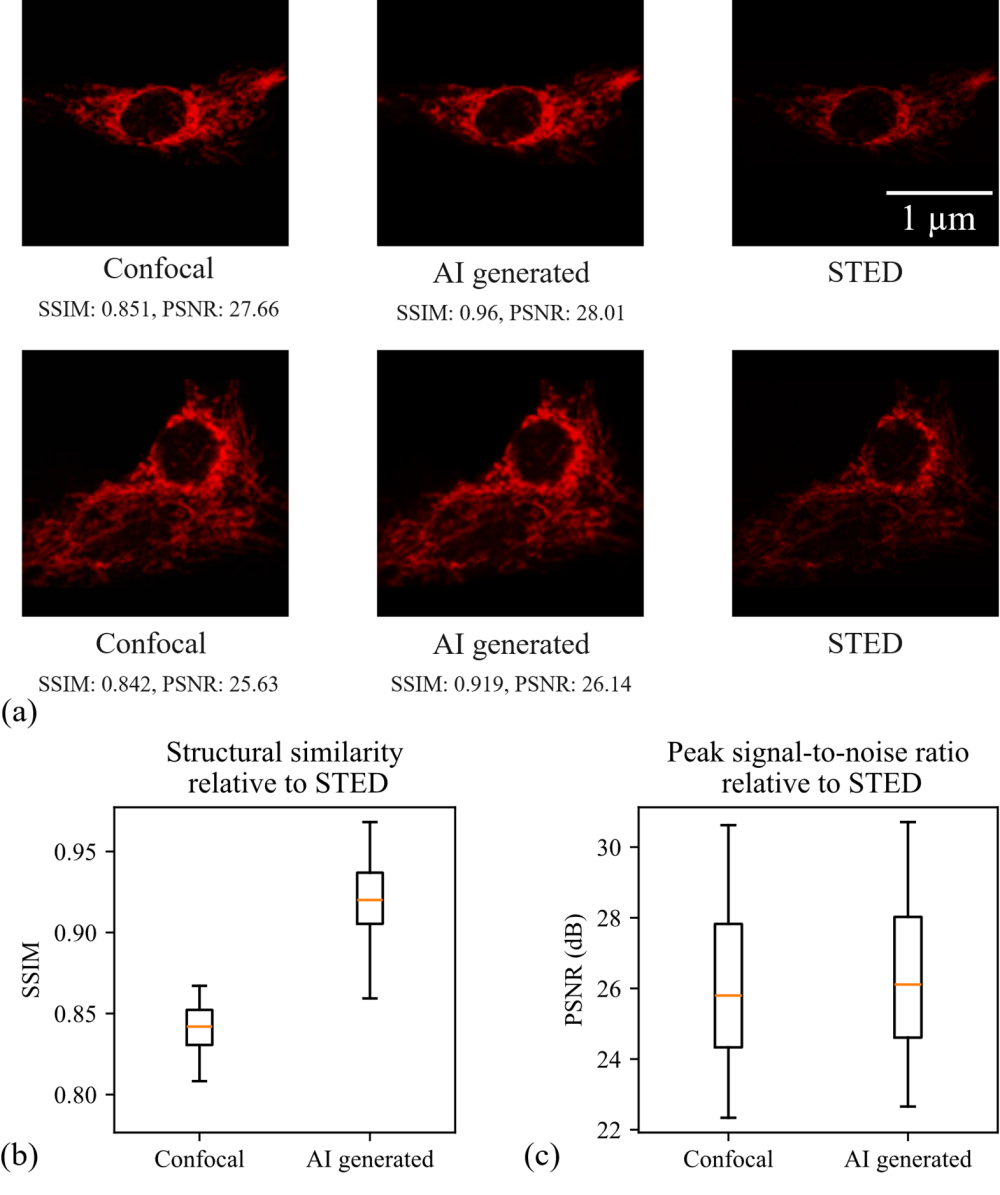
Generalization of the lightweight CycleGAN to the TOM20 dataset. (a) Representative examples of confocal, AI-generated, and STED images of mitochondria labeled in human primary M2 macrophages. Model 5 output (no fine-tuning), and STED reference for TOM20-labeled mitochondria (scale bar: 1 μm). (b) Boxplot of SSIM comparing confocal input and Model 5 output to the STED reference for 42 fields of view. (c) Boxplot of PSNR for the same dataset.

Generalization in cross-modality image transformation is particularly critical in fluorescence microscopy, where models are often trained on a specific combination of cell type, fluorophore, labeling protocol, and imaging system, but then implicitly expected to perform reliably under different experimental conditions. Prior cross-modality and super-resolution studies such as deep-learning based confocal to STED or widefield-to-super-resolved mappings and content-aware restoration networks have demonstrated impressive performance but typically within a narrowly defined domain, highlighting that shifts in sample preparation, staining density, optical alignment, or noise characteristics can degrade prediction fidelity or introduce hallucinated structures if not properly controlled [9,17]. These considerations motivate explicit tests of whether a model trained on one biological structure and staining protocol can preserve structural integrity and resolution when applied to an independent dataset and make generalization analysis a necessary component of validating cross-modality transformation frameworks for practical biomedical use. To evaluate the robustness and generalization capability of our approach beyond the ARL13B dataset, we applied Model 5 (fixed-channel lightweight CycleGAN) without fine-tuning to the independent dataset of images of mitochondria. Here, we recorded an independent dataset of 42 paired confocal and STED images of mitochondria immunolabeled for Translocase of the Outer Mitochondrial Membrane 20 (TOM20) with Abberior STAR RED in human primary M2 macrophages (see Methods 2.1, Fig. 9). On this unseen target, the translated images achieved a mean SSIM of 0.92 ± 0.028 and a mean PSNR of 26.45 ± 2.07 dB with respect to the corresponding STED images, outperforming the confocal inputs (mean SSIM 0.840 ± 0.0158; mean PSNR 26.18 ± 2.15 dB). The SSIM and PSNR distributions (boxplots) and representative examples in Fig. 9 indicate that the fixed channel model can generalize its confocal-to-STED mapping to a different protein target and staining protocol without retraining.

## 4. Conclusion

In conclusion, this study presents a detailed comparison between CycleGAN and Pix2Pix for modality transfer tasks in fluorescence microscopy, specifically transforming confocal images into STED and deconvolved STED modalities. While Pix2Pix achieved slightly higher scores, CycleGAN demonstrated competitive performance. This is especially important in biomedical imaging, where generating paired datasets is often impractical because of the high cost, time, and complexity of biological sample preparation, precise labeling, and maintaining stable imaging conditions. Our findings suggest that CycleGAN remains a viable option when paired data is limited or unavailable. This is particularly relevant for live-cell or dose-sensitive imaging, high-throughput or multi-site studies with limited access to STED, and real-time use during image acquisition in resource-constrained settings, where acquiring extensive paired super-resolution data is impractical. We also investigated the influence of generator architecture by systematically reducing the number of channels in U-Net-based models. In doing so, we compared the traditional channel-doubling policy with a fixed-channel strategy. The fixed-channel models, particularly those with simplified architectures, achieved comparable or superior performance with significantly fewer parameters. In our experiments, prioritizing lighter models does not mean accepting a substantial performance loss; instead, we show that increasing model complexity and parameter count does not monotonically improve image quality and can, in some cases, promote hallucinated structures or reduced biological fidelity. Lightweight, appropriately constrained models can therefore be advantageous in biomedical imaging, as they offer comparable quantitative performance while reducing the risk of overfitting and artifacts.

Beyond image translation, we explore GAN outputs trained on high-quality images as qualitative indicators of potential experimental or labeling inconsistencies. By training models on high-quality data and comparing their outputs to low-quality experimental results, we showed that GAN predictions can reveal subtle deviations in imaging conditions or protein expression. These observations suggest that GAN-based inference can serve as a qualitative aid for highlighting potential inconsistencies in experimental conditions or labeling, especially in high-throughput or large-scale microscopy studies. Together, these contributions highlight the utility of GANs not only for generating super-resolution images but also for guiding experimental reliability and promoting more reproducible imaging workflows.

From an application perspective, lightweight unpaired image-translation frameworks of this type are well-suited for real-time use during image acquisition and for multichannel fluorescence imaging workflows. In future work, we aim to extend these models to multi-channel image fusion and more complex live-cell microscopy scenarios.

## Supplemental information

Supplement 1 Supplementry https://doi.org/10.6084/m9.figshare.31015348

## Data Availability

Data underlying the results presented in this paper are not publicly available at this time but may be obtained from the authors upon reasonable request.

## Supplemental document

See Supplement 1 for supporting content.
