# Supplementary material for: Lightweight CycleGAN models for cross-modality image transformation and experimental quality assessment in fluorescence microscopy
Source: Biomed Opt Express. 2026 Feb 18;17(3):1476–98. doi: 10.1364/BOE.578297 (PMC13064623; doi:10.1364/BOE.578297)
Supplement: Supplementary file 1 [file boe-17-3-1476-s001.pdf]

Supplemental Document

Biomedical Optics EXPRESS

Lightweight CycleGAN models for cross-modality image transformation and experimental quality assessment in fluorescence microscopy: supplement

Mohammad Soltaninezhad,[1,2] Yashar Rouzbahani,[3,4] Jhonatan Contreras,[1,2] Francisco Paez Larios,[3,4] Paul M. Jordan,[5,6] Oliver Werz,[5,6] Rohan Chippalkatti,[7] Daniel Kwaku Abankwa,[7] Christian Eggeling,[3,4,6] and Thomas Bocklitz[1,2,*]

[1] *Department "Photonic Data Science", Leibniz Institute of Photonic Technology, Member of Leibniz Health Technologies, Member of the Leibniz Centre for Photonics in Infection Research (LPI), Jena, Germany*
[2] *Work group "Photonic Data Science", Institute of Physical Chemistry (IPC) and Abbe Center of Photonics (ACP), Friedrich Schiller University Jena, Member of the Leibniz Centre for Photonics in Infection Research (LPI), Jena, Germany*
[3] *Institute of Applied Optics and Biophysics Friedrich Schiller University Jena, Jena, Germany*
[4] *Leibniz Institute of Photonic Technologies Department of Biophysical Imaging, Jena, Germany*
[5] *Department of Pharmaceutical/Medicinal Chemistry, Institute of Pharmacy, Friedrich Schiller University Jena, 07743 Jena, Germany*
[6] *Jena Center for Soft Matter (JCSM), Friedrich Schiller University Jena, 07743 Jena, Germany*
[7] *Cancer Cell Biology and Drug Discovery group, 2 Bioinformatics Core, Department of Life Sciences and Medicine, University of Luxembourg, L-4367 Esch-sur-Alzette, Luxembourg*
[*] *thomas.bocklitz@uni-jena.de*

This supplement published with Optica Publishing Group on 18 February 2026 by The Authors under the terms of the Creative Commons Attribution 4.0 License in the format provided by the authors and unedited. Further distribution of this work must maintain attribution to the author(s) and the published article's title, journal citation, and DOI.

Supplement DOI: https://doi.org/10.6084/m9.figshare.31015348

Parent Article DOI: https://doi.org/10.1364/BOE.578297

LIGHTWEIGHT CYCLEGAN MODELS FOR CROSS-MODALITY IMAGE TRANSFORMATION AND EXPERIMENTAL QUALITY ASSESSMENT IN FLUORESCENCE MICROSCOPY: SUPPLEMENTAL DOCUMENT

S1. Image Pre-Processing:

S1.1. Mutual Information image registration

To ensure pixel-wise correspondence between modalities, confocal images were rigidly aligned to their corresponding STED or deconvolved STED (dSTED) images using a mutual information based registration procedure. Full details of the alignment algorithm, including mutual-information computation, coarse-to-fine shift search, and optimization, are implemented in Python 3.10.9 using NumPy, SciPy, and OpenCV. images are registered by maximizing mutual information computed from 64-bin joint histograms, with an initial discrete search over integer shifts followed by subpixel refinement via Powell optimization (SciPy minimize) under a bounded translation model.

Because the STED/dSTED images contain higher spatial resolution, sharper edges, and more reliable structural detail, they were used as reference images, while the confocal images served as moving images. Using STED as reference avoids bias toward the lower-resolution modality and provides a stable target for mutual-information maximization [1,2].

For each confocal–STED pair, the algorithm:

- Performed coarse MI search followed by Powell-based fine optimization.
- Applied the optimal shift (Δy, Δx) to the confocal image.
- Generated red–green overlays before and after registration.
- Saved the registered images and recorded shift values in a spreadsheet for documentation.

A representative example is shown in Fig. S1.1, demonstrating the improvement in spatial alignment after applying the optimized MI-based registration.

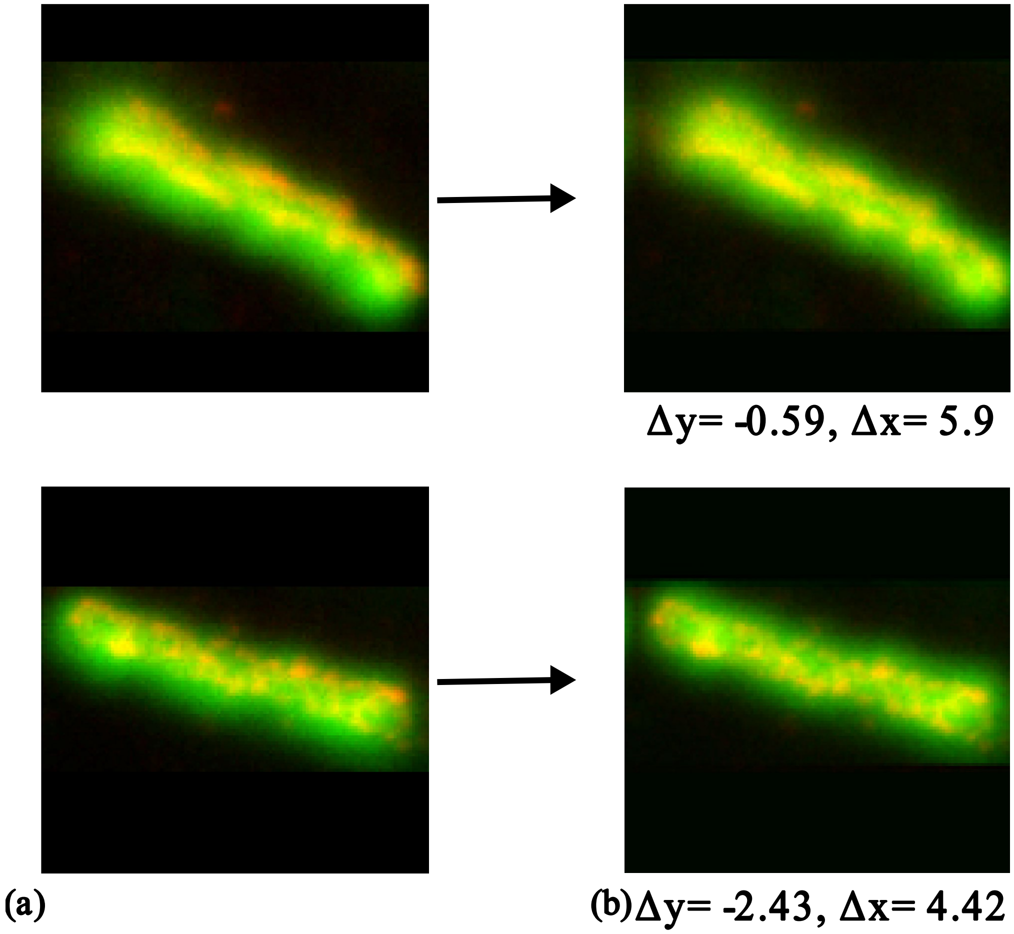

Figure S1.1: Example of mutual information image registration. The green channel shows the confocal image, and the red channel shows the STED image. (a) Before registration and (b) after registration.

S1.2 Contrast Enhancement:

We implemented this preprocessing step as an ImageJ macro (ImageJ macro language) that batch-processes raw (.msr) microscope files using the Bio-Formats importer. All images are contrast-enhanced using ImageJ's Enhance Contrast function, and the processed images are then exported as (.tif) files with informative filenames to a dedicated output directory for subsequent analysis. In ImageJ, "Enhance Contrast" performs a histogram-based intensity rescaling [3].

This improves visual contrast, standardizes the dynamic range across images, and reduces the influence of outlier pixels, while preserving relative intensity relationships in the majority of the image.

S2. Hyperparameters and Training Details:

S2.1 Hyperparameters details:

All CycleGAN models were trained under a common protocol to ensure comparability and reproducibility. We used 5-fold cross-validation, with each image appearing in the validation set exactly once, and reset the system state before experiments by clearing GPU memory, running garbage collection, and fixing all random seeds to 42 (Python, NumPy, and PyTorch). Training followed the standard CycleGAN framework with a U-Net (unet_128) generator and a PatchGAN discriminator, operating on preprocessed 128×128 patches (no additional preprocessing inside CycleGAN). Optimization was performed with Adam (initial learning rate 0.0002) and least-squares GAN loss (lsgan). Cycle-consistency losses were weighted by $\lambda\_A = \lambda\_B = 10.0$, and the identity loss by $\lambda\_identity = 0.5$. Each model was trained for 200 epochs, with a constant learning rate for the first 100 epochs followed by linear decay over the remaining 100 epochs, using a batch size of 1 and no dropout in the generators.

Table S2.1 Hyperparameters and Training Settings Used in All CycleGAN Experiments

Item	Value / Setting	Short explanation
Cross-validation	5-fold CV	Each image appears in the validation set exactly once.
Seed initialization	seed = 42 (for cross validation)	Python, NumPy, and PyTorch seeds fixed for reproducible runs.
GPU / memory reset	CUDA cache cleared	GPU memory and Python objects cleared before experiments.
Generator	UNET-128	
Discriminator	PatchGAN	
Preprocessing inside CycleGAN	None	No resizing/cropping inside CycleGAN;
Batch size	1	
Optimizer	Adam	Used for both generator and discriminator.
Learning rate	0.0002	
GAN loss type	lsgan	Least-squares GAN loss
Cycle-consistency weights	$\lambda\_A = 10$ $\lambda\_B = 10$	Strength of forward and backward cycle-consistency loss
Identity loss weight	$\lambda\_identity = 0.5$	Encourages intensity/structure preservation
Dropout	None	No dropout used in the generators

S2.2 Training details:

We monitored the adversarial training dynamics by tracking all major CycleGAN loss components: Discriminator A, Discriminator B, Generator A, Generator B, the cycle-consistency loss for domain A, the cycle-consistency loss for domain B, the identity loss for domain A, and the identity loss for domain B. For the main experiments, we fixed the random seed to 42 to ensure reproducibility. As an additional stability check, we retrained the model three times with different random seeds and summarized the domain B loss across seeds as mean ± standard deviation (Supplementary Figure S2.1), indicating that the optimization is stable and not overly sensitive to initialization. For each loss term, we then extracted the values from the training logs of all five cross-validation folds, computed the mean and standard deviation across folds, and plotted the resulting mean ± standard deviation curves. This allows us to assess convergence and training robustness and, importantly, to verify that a stable balance is maintained between generator and discriminator training: if either generators or discriminators dominate, training can become unstable or collapse, whereas jointly decreasing, smooth curves indicate a well-behaved adversarial optimization. These aggregated training curves are shown in Supplementary Figure S2.2.

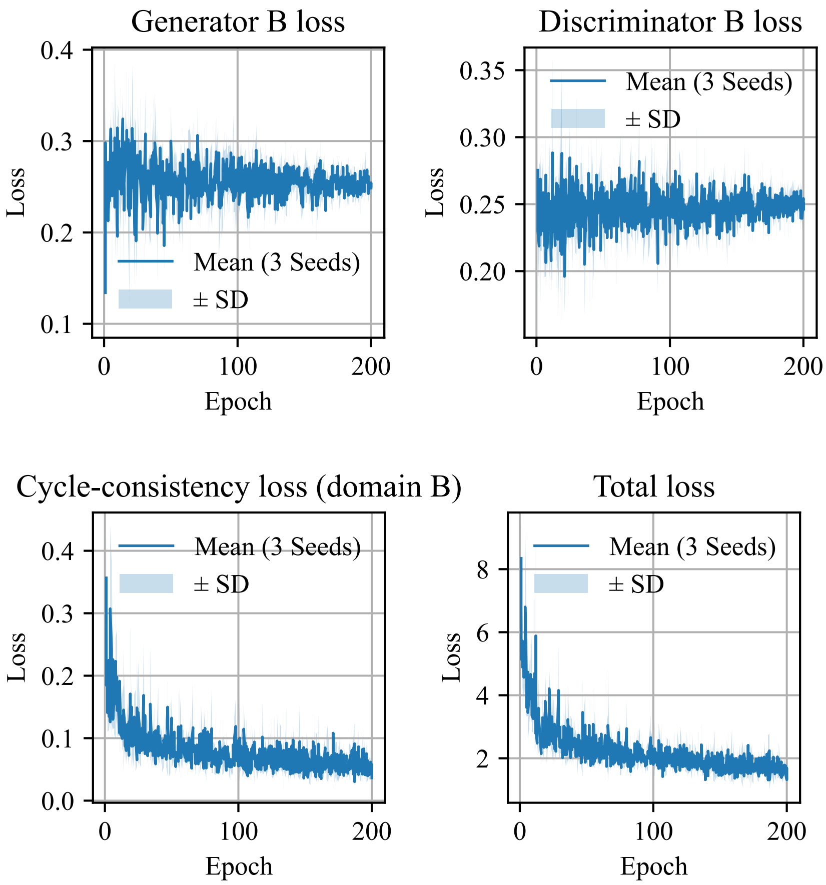

Figure S2.1. Training curves for domain B, Generator, Discriminator, cycle-consistency, and total generator loss across three random seeds, shown as mean ± standard deviation over epochs.

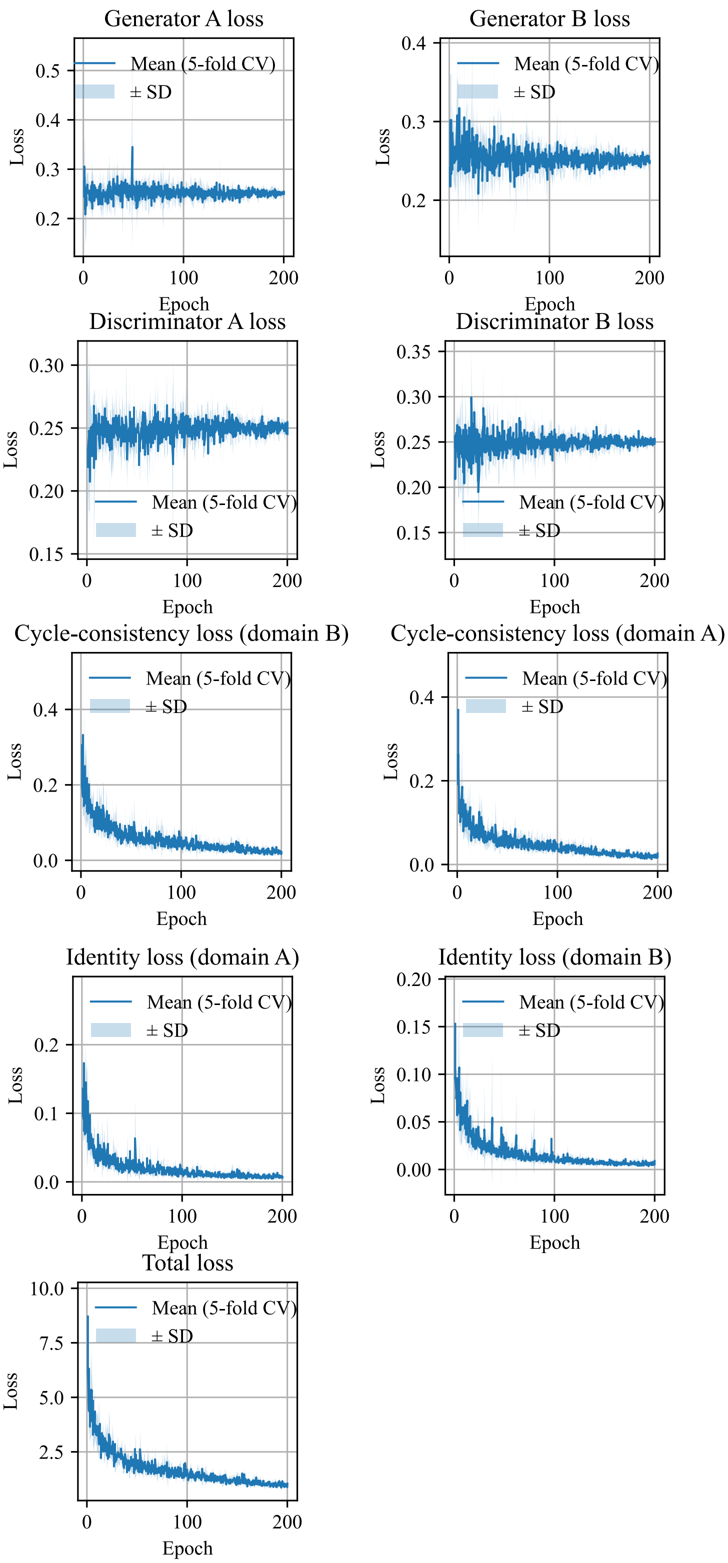

Figure S2.2. Mean ± standard deviation of CycleGAN loss component across cross-validations.

S3. Statistical Evaluation (ANOVA and Pairwise p-values)

To evaluate whether differences in SSIM and PSNR across the nine CycleGAN models were statistically significant, we performed a repeated-measures ANOVA with ImageID as the within-subject factor. This accounts for the paired structure of the dataset, where each image is reconstructed by every model. For pairwise model comparisons, we conducted paired t-tests with Holm correction to control the family-wise error rate. The analysis pipeline was implemented in Python using pandas, numpy, scipy, statsmodels, and pingouin, and the resulting p-values were exported to Excel for transparency and reproducibility. Table S3.1 reports the Holm-corrected pairwise p-values for SSIM in the confocal-to-STED task, and Table S3.2 contains the corresponding PSNR results. Tables S3.3 and S3.4 provide the analog pairwise p-value matrices for the confocal-to-deconvolved-STED (dSTED) task for SSIM and PSNR, respectively. These results quantify which model differences are statistically meaningful and which architectures yield statistically comparable reconstruction performance.

Table S3.1 — Pairwise Holm-Corrected p-Values for SSIM

(Confocal → STED)

	Model 1	Model 2	Model 3	Model 4	Model 5	Model 6	Model 7	Model 8	Model 9
**Model 1**	1	0.792885	0.248008	0.011393	1.3E-09	3.14E-48	1	6.25E-07	0.017932
**Model 2**	0.792885	1	0.011362	0.001172	3.02E-10	1.33E-45	1	0.000547	0.164885
**Model 3**	0.248008	0.011362	1	0.792885	0.000168	5.7E-43	1	1.71E-11	0.000157
**Model 4**	0.011393	0.001172	0.792885	1	0.089408	4.35E-37	0.405552	1.19E-13	9.12E-06
**Model 5**	1.3E-09	3.02E-10	0.000168	0.089408	1	3.02E-30	0.001491	3.11E-24	1.17E-16
**Model 6**	3.14E-48	1.33E-45	5.7E-43	4.35E-37	3.02E-30	1	1.02E-38	2.65E-55	2.54E-42
**Model 7**	1	1	1	0.405552	0.001491	1.02E-38	1	0.008916	0.248008
**Model 8**	6.25E-07	0.000547	1.71E-11	1.19E-13	3.11E-24	2.65E-55	0.008916	1	1
**Model 9**	0.017932	0.164885	0.000157	9.12E-06	1.17E-16	2.54E-42	0.248008	1	1

Table S3.2 — Pairwise Holm-Corrected p-Values for PSNR

(Confocal → STED)

	**Model 1**	**Model 2**	**Model 3**	**Model 4**	**Model 5**	**Model 6**	**Model 7**	**Model 8**	**Model 9**
**Model 1**	1	0.20669	0.198272	0.019377	0.000586	1	0.075983	1	1
**Model 2**	0.20669	1	1	1	7.45E-08	0.038783	1	1	1
**Model 3**	0.198272	1	1	0.89981	3.25E-09	0.012804	1	1	1
**Model 4**	0.019377	1	0.89981	1	1.03E-13	1.73E-06	1	0.030535	0.849292
**Model 5**	0.000586	7.45E-08	3.25E-09	1.03E-13	1	0.226734	3.62E-10	6.27E-05	0.000141
**Model 6**	1	0.038783	0.012804	1.73E-06	0.226734	1	1.47E-07	0.060022	0.026048
**Model 7**	0.075983	1	1	1	3.62E-10	1.47E-07	1	0.0044	0.620129
**Model 8**	1	1	1	0.030535	6.27E-05	0.060022	0.0044	1	1
**Model 9**	1	1	1	0.849292	0.000141	0.026048	0.620129	1	1

Table S3.3 — Pairwise Holm-Corrected p-Values for SSIM

(Confocal → Deconvolved STED)

	**Model 1**	**Model 2**	**Model 3**	**Model 4**	**Model 5**	**Model 6**	**Model 7**	**Model 8**	**Model 9**
**Model 1**	1	0.016324	1	0.000443	0.995563	0.000175	1	0.001116	0.025832
**Model 2**	0.016324	1	6.12E-05	8.29E-10	4.53E-06	6.18E-11	0.000818	7.49E-10	5.18E-08
**Model 3**	1	6.12E-05	1	0.059365	1	0.004084	1	0.00636	0.09109
**Model 4**	0.000443	8.29E-10	0.059365	1	0.037101	1	0.000199	1	1
**Model 5**	0.995563	4.53E-06	1	0.037101	1	0.001935	0.231407	0.005848	0.025549
**Model 6**	0.000175	6.18E-11	0.004084	1	0.001935	1	5.11E-06	1	1
**Model 7**	1	0.000818	1	0.000199	0.231407	5.11E-06	1	6.25E-07	0.000175
**Model 8**	0.001116	7.49E-10	0.00636	1	0.005848	1	6.25E-07	1	1
**Model 9**	0.025832	5.18E-08	0.09109	1	0.025549	1	0.000175	1	1

Table S3.4 — Pairwise Holm-Corrected p-Values for PSNR (Confocal → Deconvolved STED)

	Model 1	Model 2	Model 3	Model 4	Model 5	Model 6	Model 7	Model 8	Model 9
**Model 1**	1	0.033264	0.089676	5.49E-18	3.55E-11	4.49E-17	1.09E-16	2.08E-10	2.61E-21
**Model 2**	0.033264	1	1.15E-06	2.91E-20	1.41E-17	2.19E-21	1.82E-22	6.08E-15	6.42E-24
**Model 3**	0.089676	1.15E-06	1	2.09E-17	1.34E-10	1.27E-15	1.53E-18	6.94E-10	2.49E-21
**Model 4**	5.49E-18	2.91E-20	2.09E-17	1	0.000664	1	1	0.010392	0.000348
**Model 5**	3.55E-11	1.41E-17	1.34E-10	0.000664	1	0.002698	0.000123	1	2.69E-11
**Model 6**	4.49E-17	2.19E-21	1.27E-15	1	0.002698	1	1	0.059186	5.96E-05
**Model 7**	1.09E-16	1.82E-22	1.53E-18	1	0.000123	1	1	0.018776	5.94E-05
**Model 8**	2.08E-10	6.08E-15	6.94E-10	0.010392	1	0.059186	0.018776	1	3.24E-09
**Model 9**	2.61E-21	6.42E-24	2.49E-21	0.000348	2.69E-11	5.96E-05	5.94E-05	3.24E-09	1

S4. Deviation Maps and Anomaly Scores

To further evaluate the per-pixel agreement between each model output and the ground-truth STED image, we computed deviation maps by taking the absolute intensity difference between the generated and ground-truth for every pixel and visualized the result as a heatmap. From each deviation map, we also calculated a mean anomaly score, defined as the average absolute deviation across all pixels. This provides a compact, quantitative measure of how closely each model reproduces the ground-truth image. Figure S4.1 and Figure S4.2 show representative examples for respectively confocal to STED and confocal to deconvolved STED, including some models' generated images alongside their corresponding deviation heatmaps. The anomaly score for each model, computed with respect to the ground truth, is also summarized to enable direct comparison across all architectures.

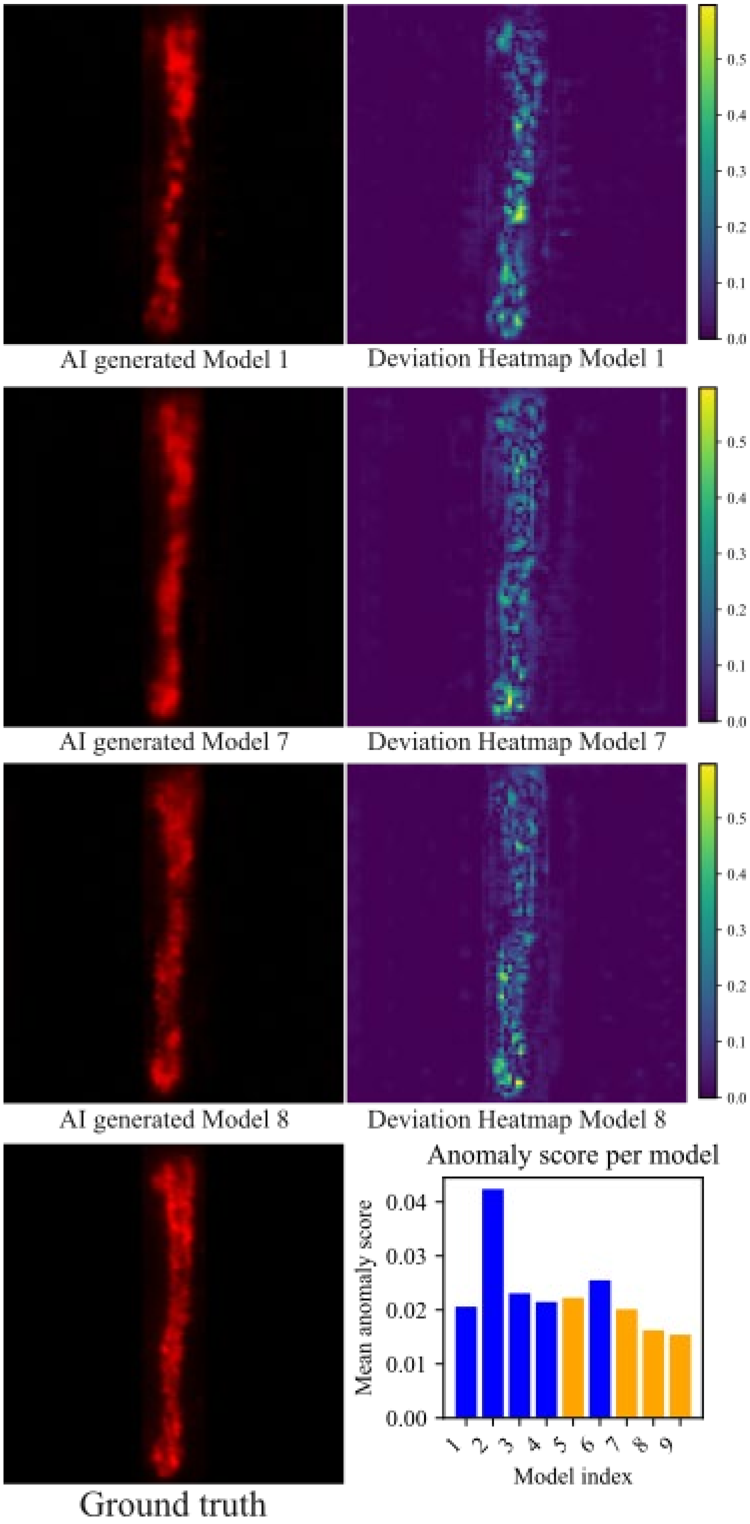

Figure S4.1 Confocal to STED modality transfer: deviation heatmaps and anomaly score graph across models

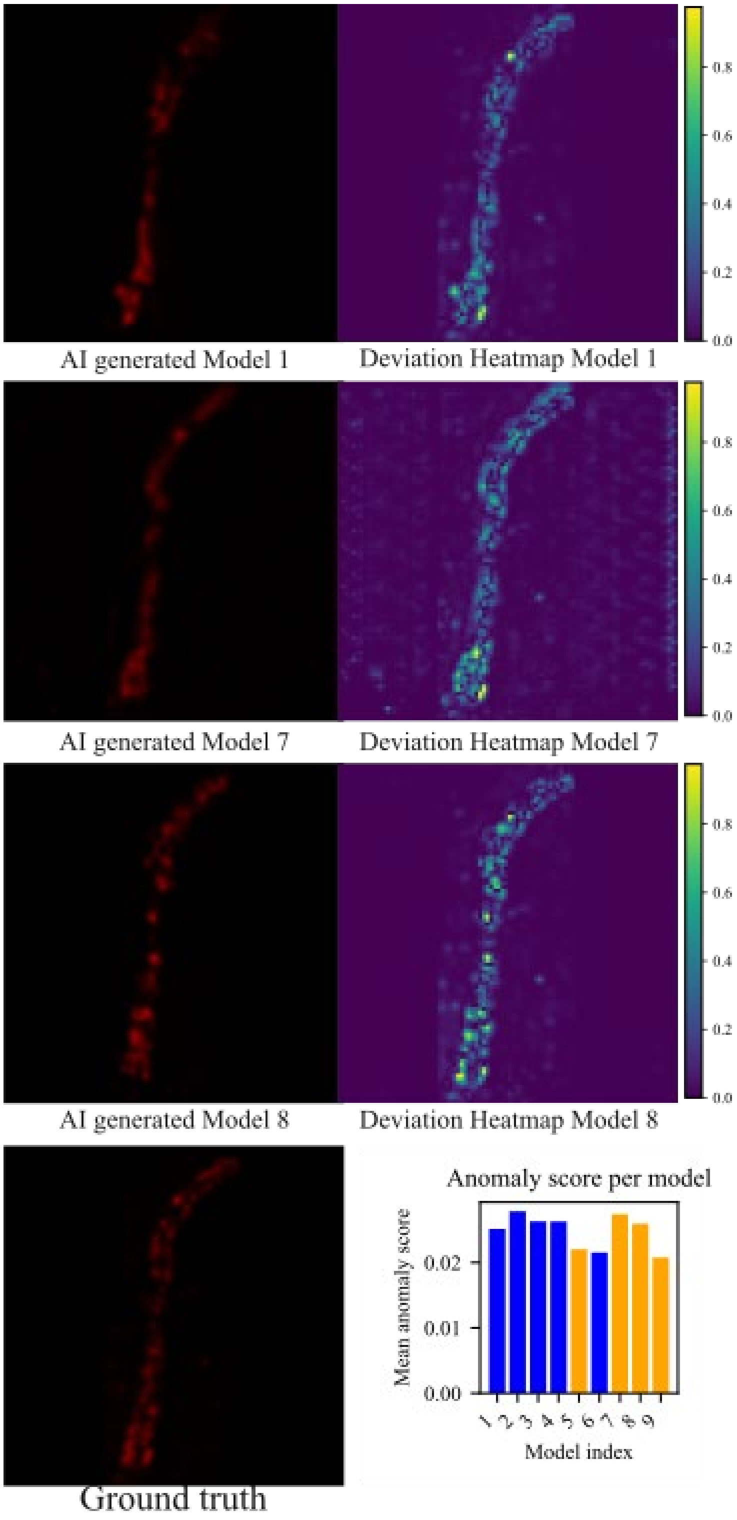

Figure S4.2 Confocal to Deconvolved STED modality transfer: deviation heatmaps and anomaly score graph across models

References

1. https://docs.scipy.org/doc/scipy/reference/generated/scipy.ndimage.shift.html
2. https://docs.scipy.org/doc/scipy/reference/generated/scipy.optimize.minimize.html
3. C. A. Schneider, W. S. Rasband, and K. W. Eliceiri, "NIH Image to ImageJ: 25 years of image analysis," Nat Methods 9, 671-675 (2012).
